# The combined impact of meconium stained amniotic fluid and small for gestational age on delivery outcomes

**DOI:** 10.1007/s00404-025-07995-8

**Published:** 2025-03-10

**Authors:** Gal Cohen, Adi Shilony, Reut Batia Amrami, Tal Biron-Shental, Michal Kovo, Hanoch Schreiber

**Affiliations:** 1 Department of Obstetrics and Gynecology, Meir Medical Center, 59 Tchernichovsky St, 44281 Kfar SabaKfar Saba, Israel; 2 School of Medicine, Faculty of Medical and Health Sciences, Tel Aviv University, Tel Aviv, Israel

**Keywords:** Cesarean delivery, Adverse neonatal outcomes, Fetal growth restriction, Meconium stained amniotic fluid

## Abstract

**Purpose:**

To assess the delivery complications in neonates with meconium stained amniotic fluid (MSAF) and small for gestational age (SGA) birthweight.

**Methods:**

The medical records of all term, singleton deliveries during 2014–2021 were reviewed. Obstetric characteristics and neonatal outcomes were evaluated among the following groups: SGA neonates with MSAF (SGA-MSAF group), SGA neonates without MSAF (SGA group), appropriate for gestational age (AGA) neonates with MSAF (AGA-MSAF group) and AGA without MSAF (AGA group).

**Results:**

A total of 44,911 deliveries were included in the study, with 673 in the SGA-MSAF group, 2,762 in the SGA group, 6,958 in the AGA-MSAF group, and 34,518 in the AGA group. The SGA-MSAF group exhibited higher rates of nulliparity and hypertensive disorders compared to the SGA, AGA-MSAF, and AGA groups (*p* ≤ 0.001). Oligohydramnios, labor induction, vacuum extractions (VE), and intrapartum cesarean deliveries (CD) were significantly more frequent in the SGA-MSAF group compared to the SGA, AGA-MSAF, and AGA groups (*p* ≤ 0.003). The SGA-MSAF group had the highest rates of adverse composite neonatal outcomes compared to the SGA, AGA-MSAF, and AGA groups (*p* < 0.001). Multivariable logistic regression, adjusted for confounders, revealed increased ORs for the adverse neonatal composite outcome, VE, VE due to NRFHR, intrapartum CD, and CD due to NRFHR, in the presence of MSAF or SGA, and mostly when both risk factors were present (*p* ≤ 0.002).

**Conclusion:**

Deliveries complicated with MSAF and SGA were associated with increased obstetric complications compared to each alone. Clinicians should be aware of this and manage labor accordingly.

## What does this study add to the clinical work

**Table:** 

Deliveries complicated with both MSAF and SGA are associated with higher obstetric complications compared to either condition alone. Awareness of this association should guide clinicians in optimizing labor management.	

## Introduction

The occurrence of meconium-stained amniotic fluid (MSAF) increases with gestational age, reaching 5–20% by the time of delivery [1]. Although intrauterine defecation is thought to be a physiological process, it has been associated with hypoxia, intraamniotic infection and inflammation, and post-term pregnancies [1]. MSAF carries neonatal risks as fetal acidemia, respiratory distress, sepsis, encephalopathy, seizures and cerebral palsy [1], [2], [3], [4], [5] All are thought to be explained by the inflammatory processes in fetal organs exposed to MSAF, along with the heightened risk of microbial invasion into the amniotic cavity [6, 7]. Obstetric risks have also been reported, including higher rates of vacuum extractions (VE), cesarean deliveries (CD), and intrapartum non-reassuring fetal heart rate (NRFHR) patterns [8]. Although associated with short-term perinatal complications, MSAF might have a protective role against long-term respiratory morbidity [9], dermatitis [10] and infectious morbidity in the offspring. [11]

Fetal growth restriction (FGR) has been associated with neonatal acidemia, perinatal adverse outcomes and increased mortality [12, 13]. The International Society of Ultrasound in Obstetrics and Gynecology [14] and the Delphi consensus regarding fetal growth restriction [15], differentiate FGR from small for gestational age fetuses (SGA) who are considered constitutionally small and generally at low risk for adverse outcomes. Data regarding the perinatal risks associated with SGA birthweight are inconclusive. Akerman et al. [16] compared umbilical cord pH between SGA and appropriate for gestational age (AGA) neonates and concluded that SGA have an intact ability to develop lacticemia in response to hypoxia, while another study excluded this risk [17].

MSAF during delivery is suggestive of fetal distress. Given that SGA fetuses are presumed to have a reduced ability to manage stress, they may experience poorer neonatal outcomes compared to AGA fetuses when MSAF is present. An evaluation of SGA compared to AGA neonates exhibiting MSAF has not been performed yet. This study examined the combined impact of MSAF and SGA on delivery outcomes.

## Methods

This retrospective cohort study included women who delivered in a single tertiary care medical center from January 1, 2014 to September 1, 2021. Term singleton pregnancies presenting with SGA neonates and MSAF (SGA-MSAF group), were compared to three control groups: SGA neonates without MSAF (SGA group), AGA neonates with MSAF (AGA-MSAF group) and AGA neonates without MSAF (AGA group). SGA was defined as neonatal birthweight below the tenth percentile diagnosed according to local birthweight charts [18].

To assure neonatal adverse outcomes were associated only with SGA and/or MSAF, we excluded all preterm deliveries, pregnancies with known fetal chromosomal or structural anomalies and FGR fetuses, as defined by the Delphi consensus regarding fetal growth restriction[15] as weights below the third percentile or below the tenth percentile with Doppler abnormalities. Multifetal pregnancies, stillbirths and cases with missing data were also excluded.

The medical records of the SGA MSAF group were compared to controls in terms of basic maternal features, labor and delivery characteristics, and obstetric outcomes. First, the SGA MSAF group was compared to all controls together, then to each control group separately, to evaluate whether there was an additive risk in the presence of both SGA and MSAF compared to each of the two alone.

The primary outcome was the composite neonatal adverse outcome, which included one or more of the following: hypoglycemia, need for phototherapy, hypothermia, meconium aspiration syndrome, need for non-invasive ventilation, sepsis, hypoxic ischemic encephalopathy or seizures. The secondary outcomes were mode of delivery and other neonatal outcomes (5-min Apgar scores < 7 and umbilical cord pH < 7.1).

### Data collection

Data were retrieved using the electronic maternal databases of the obstetric triage unit and the delivery room, which were cross-tabulated with data from the neonatal unit and the Neonatal Intensive Care Unit (NICU).

The following information was collected:

1. Maternal demographics and medical history: age, gravidity, parity, history of CD, body mass index (BMI, kg/m^2^), smoking, diabetes mellitus (DM) including pregestational and gestational diabetes mellitus [19], chronic hypertension and gestational hypertensive disorders [20].
2. Ultrasound at presentation: presence of poly- or oligohydramnios[21] and estimated fetal weight.
3. Delivery characteristics: gestational age (GA) at delivery, use of epidural anesthesia, intrapartum fever (maternal temperature ≥ 38°C during delivery or up to 24 h after delivery), amniotic fluid color, mode of delivery (normal vaginal delivery [NVD], VE or CD) and indication for VE or CD.
4. All neonates were evaluated by a pediatrician immediately after delivery, or by a neonatologist if their birthweight was < 2500 g. Neonatal outcomes collected included Apgar scores at 5 min, umbilical cord pH, neonatal birthweight (SGA, AGA [18]), NICU admission, hypoglycemia (blood glucose < 40 mg/dL), need for phototherapy, hypothermia, meconium aspiration syndrome, need for non-invasive ventilation, sepsis, hypoxic ischemic encephalopathy or seizures.

### Ethics approval

The study was approved by the Institutional Ethics Committee in September 2021, approval number 0282-21-MMC. Due to the retrospective nature of the data collection, the Ethics Committee exempted the authors from obtaining individual informed consent.

### Statistical analysis

Categorical data were compared using Chi-square or Fisher’s exact test, each when appropriate. Continuous variables were tested for normal distribution using the Shapiro–Wilk test, and comparisons between groups with a normal distribution were compared using t-test. Multivariable logistic regression analysis was performed to evaluate the adjusted risk for delivery complications in the AGA-MSAF, SGA and SGA-MSAF groups compared to controls (AGA group). Factors included in this analysis were independent variables that were found significantly different between groups (p < 0.05) in the univariate analysis. Results were reported as adjusted odds ratios (aOR) and 95% confidence intervals (CI). A probability value of < 0.05 was considered significant. All analyses were performed using SPSS-29 (IBM Corp., Armonk, NY, USA).

## Results

**Fig. 1:**
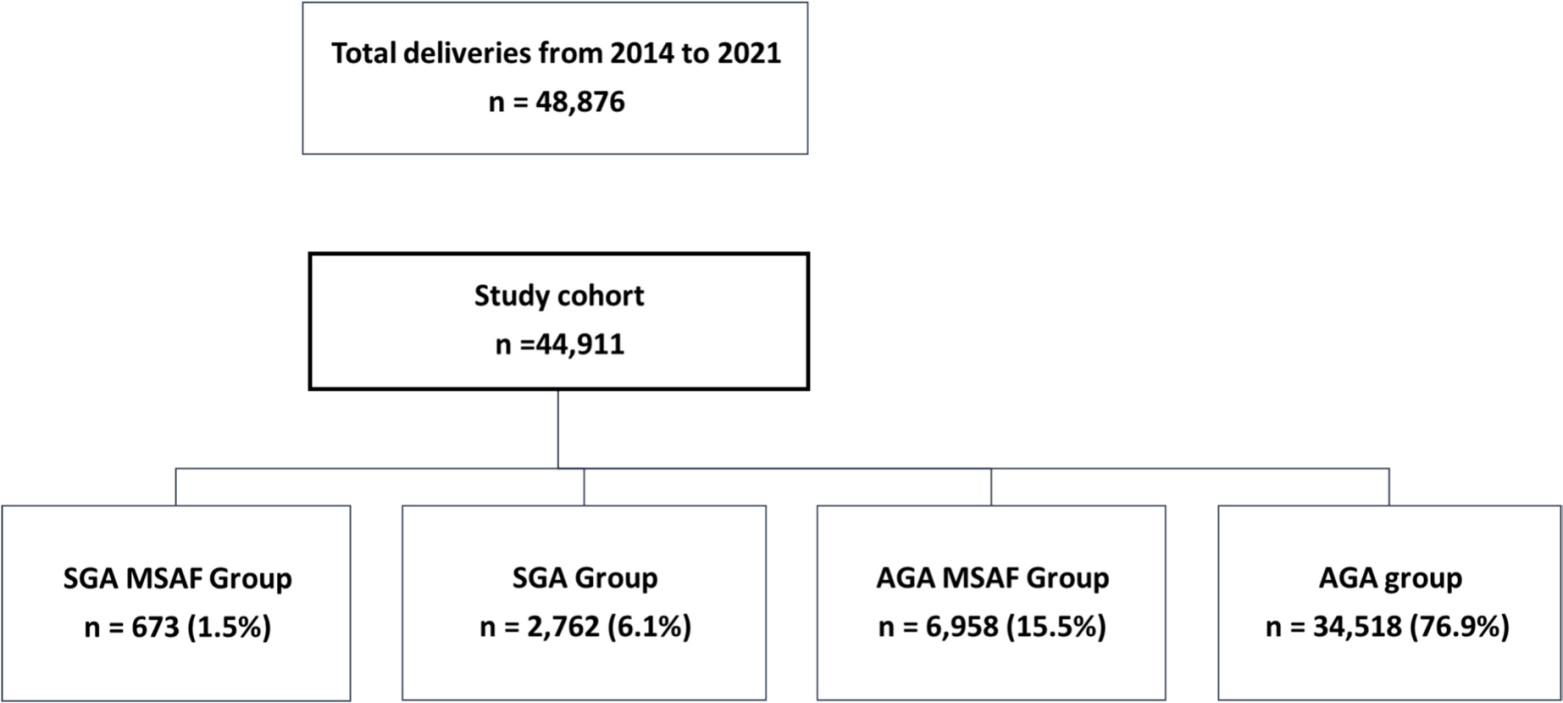
Flowchart describing the study population. *FGR* fetal growth restriction, *SGA* small for gestational age, *AGA* appropriate for gestational age, MSAF meconium stained amniotic fluid

During the study period, 48,876 women delivered in our institution, of whom 44,911 met the study inclusion criteria (Fig. 1). Among them, 673 (1.5%) had SGA neonates with MSAF, and were compared to 3 control groups: SGA neonates without MSAF (2,762, 6.1%), AGA neonates with MSAF (6,958, 15.5%) and AGA neonates without MSAF (34,518, 76.9%).

### Maternal obstetric characteristics

Nulliparity was more common in the SGA MSAF group compared to the controls (56.6% in the SGA MSAF group, vs. 46.9%, 39.9% and 30.4% in the SGA, AGA MSAF and AGA groups, respectively, p < 0.001). Lower BMI and lower rates of obesity characterized the SGA MSAF group as compared to both AGA groups (BMI 23.3 ± 3.4 in the SGA MSAF group vs. 24.2 ± 3.8 and 23.9 ± 3.6 in the AGA MSAF and AGA groups respectively, p < 0.001. Obesity rates were 4.3% the SGA MSAF group vs. 6.9% and 6.2% in the AGA MSAF and AGA groups respectively, *p* = 0.010, *p* = 0.043 respectively). Rates of maternal smoking and hypertensive disorders were higher in the SGA MSAF group compared to both AGA MSAF and AGA groups (9.7% vs. 5.8% and 5.1% respectively, *p* < 0.001; and 6.7% vs. 3.4% and 4.1% respectively, *p* < 0.001). A history of CD was less common in the SGA MSAF group compared to the SGA and AGA groups (7.0% vs. 10.5% and 11.8% respectively, *p* = 0.006, *p* < 0.001 respectively). DM rates were similar among all study groups (Table 1). Gestational ages had minor differences between groups (data not shown).

**Table 1:** Maternal characteristics in the SGA MSAF group compared to controls

Variable	SGA + MSAF n = 673	All three control groups n = 44,238	P-value	SGA w/o MSAF n = 2,762		P-value	AGA + MSAF n = 6,958	P-value	AGA w/o MSAF n = 34,518	P-value
Maternal age (years)	30.8 ± 5.9	31.1 ± 5.3	0.099		30.8 ± 5.4	0.767	31.3 ± 5.2	0.029	31.1 ± 5.3	0.102
Smoking	65 (9.7%)	2397 (5.4%)	< 0.001		220 (8.0%)	0.153	407 (5.8%)	< 0.001	1770 (5.1%)	< 0.001
Nulliparity	381 (56.6%)	14,553 (32.9%)	< 0.001		1294 (46.9%)	< 0.001	2773 (39.9%)	< 0.001	10,486 (30.4%)	< 0.001
BMI (kg/m^2^)	23.3 ± 3.4	23.9 ± 3.7	< 0.001		23.2 ± 3.6	0.455	24.2 + 3.8	< 0.001	23.9 ± 3.6	< 0.001
Obesity (BMI > 30)	29 (4.3%)	2732 (6.2%)	0.045		112 (4.1%)	0.766	480 (6.9%)	0.010	2140 (6.2%)	0.043
Previous Cesarean Delivery	47 (7.0%)	4826 (10.9%)	< 0.001		289 (10.5%)	0.006	477 (6.9%)	0.897	4060 (11.8%)	< 0.001
DM	62 (9.2%)	4775 (10.8%)	0.189		228 (8.3%)	0.423	647 (9.3%)	0.941	3900 (11.3%)	0.090
HTN/PE	45 (6.7%)	1839 (4.2%)	0.001		196 (7.1%)	0.708	237 (3.4%)	< 0.001	1406 (4.1%)	< 0.001

Continuous variables are presented as mean ± SD and categorical variables as n (%);

*MSAF* meconium stained amniotic fluid, *DM* includes pre-gestational and gestational diabetes mellitus, *HTN* hypertension, *PE* preeclampsia

### Delivery characteristics

Induction of labor was more common in the SGA MSAF group compared to both AGA MSAF and AGA groups (*p* < 0.001 and *p* = 0.002, respectively). Epidural use and intrapartum fever were more common in both MSAF groups compared to the non-MSAF groups (*p* = 0.002 and *p* = 0.007 respectively for epidural use; *p* < 0.001 for intrapartum fever). Oligohydramnios was more common in the SGA MSAF group compared to both AGA MSAF and AGA groups (5.5% vs. 1.8% and 2.0% respectively, *p* < 0.001) (Table 2).

**Table 2:** Delivery characteristics in the SGA MSAF group compared to controls

Variable	SGA + MSAF n = 673	All three control Groups n = 44,238	P-value	SGA w/o MSAF n = 2,762		P-value	AGA + MSAF n = 6,958	P-value	AGA w/o MSAF n = 34,518	P-value
Induction of labor	170, (27.8%)	8911 (22.7%)	0.003		707, (29.1%)	0.506	1374, (20.9%)	< 0.001	6830, (22.5%)	0.002
Epidural anesthesia	451, (67.0%)	27,896 (63.1%)	0.035		1675, (60.6%)	0.002	4857, (69.8%)	0.133	21,364, (61.9%)	0.007
Intrapartum fever	37, (5.5%)	1280 (2.9%)	< 0.001		52, (1.9%)	< 0.001	375, (5.4%)	0.906	853, (2.5%)	< 0.001
Polyhydramnios	0, (0.0%)	187 (0.4%)	0.091		3, (0.1%)	1.000	28, (0.4%)	0.170	156, (0.5%)	0.080
Oligohydramnios	37, (5.5%)	952 (2.2%)	< 0.001		146, (5.3%)	0.829	128, (1.8%)	< 0.001	678, (2.0%)	< 0.001
Mode of delivery										
NVD	416 (61.8%)	33,667 (76.1%)	< 0.001		1930, (69.9%)	< 0.001	5188, (74.6%)	< 0.001	26,549 (76.9%)	< 0.001
VE	119 (17.7%)	3685 (8.3%)	< 0.001		308, (11.2%)	< 0.001	837 (12.0%)	< 0.001	2540 (7.4%)	< 0.001
VE due to NRFHR	107 (15.9%)	2816 (6.4%)	< 0.001		260 (9.4%)	< 0.001	653 (9.4%)	< 0.001	1904 (5.5%)	< 0.001
CD	138, (20.5%)	6850 (15.5%)	< 0.001		521, (18.9%)	0.332	927 (13.3%)	< 0.001	5402 (15.7%)	0.001
Intrapartum CD	135 (20.1%)	3768 (8.5%)	< 0.001		345 (12.5%)	< 0.001	849 (12.2%)	< 0.001	2574 (7.5%)	< 0.001
CD due to NRFHR	112 (16.6%)	1770 (4.0%)	< 0.001		221 (8.0%)	< 0.001	486 (7.0%)	< 0.001	1063 (3.1%)	< 0.001

*MSAF* meconium stained amniotic fluid, *NVD* normal vaginal delivery, *VE* vacuum extraction, *CD* Cesarean delivery, *NRFHR* non-reassuring fetal heart rate

### Obstetric outcomes

Intrapartum CD rates were highest in the SGA MSAF group compared to the controls (20.1% vs. 12.5%, 12.2% and 7.5% in the SGA, AGA MSAF and AGA groups, respectively; *p* < 0.001). Specifically, CD due to NRFHR (16.6% vs. 8.0%, 7.0% and 3.1% respectively, *p* < 0.001) occurred more often.

VE rates were higher in the SGA MSAF group (17.7%) compared to the controls (11.2%, 12.0% and 7.4% in the SGA, AGA MSAF and AGA groups), specifically VE due to NRFHR (15.9% in the SGA MSAF group, vs. 9.4%, 9.4% and 5.5% respectively, *p* < 0.001) (Table 2).

### Neonatal outcomes

Composite adverse neonatal outcome was more common in the SGA MSAF group compared to the controls (12.6% in the SGA MSAF group, vs. 9.2%, 7.6% and 6.0% in the SGA, AGA MSAF and AGA groups, respectively, *p* = 0.022, *p* < 0.001, *p* < 0.001 respectively). NICU hospitalization was more common in SGA MSAF neonates compared to AGA neonates (1.0% vs. 0.5%, *p* = 0.035; Table 3).

**Table 3:** Neonatal outcomes in the SGA MSAF group compared to controls

Variable	SGA + MSAF n = 673	All 3 control groups n = 44,238	P-value	SGA w/o MSAF n = 2,762	P-value	AGA + MSAF n = 6,958	P-value	AGA w/o MSAF n = 34,518	P-value
Neonatal birthweight, (g) mean ± SD	2619 ± 216	3293 ± 420	< 0.001	2555 ± 232	< 0.001	3395 ± 370	< 0.001	3332 ± 383	< 0.001
Apgar < 7 at 5 min	5 (0.7%)	128 (0.3%)	0.032	9 (0.3%)	0.129	37 (0.5%)	0.480	82 (0.2%)	0.009
Cord pH < 7.1	18 (4.9%)	499 (4.8%)	0.895	45 (5.0%)	0.966	138 (5.1%)	0.863	316 (4.6%)	0.777
NICU hospitalization	7 (1.0%)	295 (0.7%)	0.240	37 (1.3%)	0.536	95 (1.4%)	0.483	163 (0.5%)	0.035
Meconium Aspiration syndrome	1 (0.2%)	29 (0.1%)	0.370	0 (0.0%)	0.182	29 (0.5%)	0.296	0 (0.0%)	0.107
Composite neonatal outcome*	62 (12.6%)	2416 (6.4%)	< 0.001	202 (9.2%)	0.022	427 (7.6%)	< 0.001	1787 (6.0%)	< 0.001

*NICU* Neonatal intensive care unit

^*^Including one or more of the outcomes: hypoglycemia, need for phototherapy, hypothermia, meconium aspiration syndrome, need for non-invasive ventilation, sepsis, hypoxic ischemic encephalopathy or seizures

### Multivariable regression analysis

Applying multivariable logistic regression analysis, adjusted for confounders, revealed increased odds ratio (OR) risks for the composite adverse neonatal outcome, VE, VE due to NRFHR, intrapartum CD, and CD due to NRFHR, in the presence of MSAF or SGA, and mostly when both risk factors were present. The aOR for the composite adverse neonatal outcome was 1.33 (95% CI 1.18–1.50) in the AGA MSAF group, 1.31 (95% CI 1.10–1.55) in the SGA group and 2.09 (95% CI 1.55–2.81) in the SGA MSAF group, *p* ≤ 0.001 for all.

The aOR for VE was 1.30 (95% CI 1.19–1.42) in the AGA MSAF group, 1.21 (95% CI 1.05–1.39) in the SGA group and 1.73 (95% CI 1.39–2.17) in the SGA MSAF group; *p* ≤ 0.002 for all. The aOR for VE due to NRFHR was 1.35 (95% CI 1.22–1.49) in the AGA MSAF group, 1.40 (95% CI 1.21–1.61) in the SGA group and 2.14 (95% CI 1.70–2.70) in the SGA MSAF group; *p* < 0.001 for all.

The aOR for intrapartum CD was 1.67 (95% CI 1.51–1.85) in the AGA MSAF group, 1.25 (95% CI 1.07–1.46) in the SGA group and 2.02 (95% CI 1.58–2.58) in the SGA MSAF group; *p* ≤ 0.001 for all. The aOR for intrapartum CD due to NRFHR was 1.92 (95% CI 1.69–2.19) in the AGA MSAF group, 1.95 (95% CI 1.63–2.34) in the SGA group and 3.37 (95% CI 2.59–4.39) in the SGA MSAF group; *p* < 0.001 for all (Table 4).

**Table 4:** Multivariable Logistic Regression Analysis- Evaluation of the risk for adverse obstetric outcomes

Risk for composite adverse neonatal outcome (compared to an AGA fetus without meconium)			
	adjusted Odds Ratio	95% Confidence Interval	P Value
AGA w/o MSAF	1.00	-	-
AGA + MSAF	1.33	1.18–1.50	< 0.001
SGA w/o MSAF	1.31	1.10–1.55	0.002
SGA + MSAF	2.09	1.55–2.81	< 0.001

Risk for vacuum extraction (compared to an AGA fetus without meconium)			
	adjusted Odds Ratio	95% Confidence Interval	P Value
AGA w/o MSAF	1.00	-	-
AGA + MSAF	1.30	1.19–1.42	< 0.001
SGA w/o MSAF	1.21	1.05–1.39	0.002
SGA + MSAF	1.73	1.39–2.17	< 0.001

Risk for vacuum extraction due to NRFHR (compared to an AGA fetus without meconium)			
	adjusted Odds Ratio	95% Confidence Interval	P Value
AGA w/o MSAF	1.00	-	-
AGA + MSAF	1.35	1.22–1.49	< 0.001
SGA w/o MSAF	1.40	1.21–1.61	< 0.001
SGA + MSAF	2.14	1.70–2.70	< 0.001

Risk for intrapartum cesarean delivery (compared to an AGA fetus without meconium)			
	adjusted Odds Ratio	95% Confidence Interval	P Value
AGA w/o MSAF	1.00	-	-
AGA + MSAF	1.67	1.51–1.85	< 0.001
SGA w/o MSAF	1.25	1.07–1.46	0.001
SGA + MSAF	2.02	1.58–2.58	< 0.001

Risk for cesarean delivery due to NRFHR (compared to an AGA fetus without meconium)			
	adjusted Odds Ratio	95% Confidence Interval	P Value
AGA w/o MSAF	1.00	-	-
AGA + MSAF	1.92	1.69–2.19	< 0.001
SGA w/o MSAF	1.95	1.63–2.34	< 0.001
SGA + MSAF	3.37	2.59–4.39	< 0.001

*MSAF* meconium stained amniotic fluid*, NRFHR* non-reassuring fetal heart rate, *w/o* without. Multivariable regression model was adjusted for confounders that were significantly different between groups in the univariate analysis, or were thought to have an effect on the outcomes evaluated. These factors included: maternal age, gestational age, smoking, nulliparity, obesity, hypertensive disorders, diabetes mellitus, history of cesarean delivery, oligohydramnios, induction of labor, epidural anesthesia and intrapartum fever. Results are reported as adjusted odds ratios and 95% confidence intervals

## Discussion

### Principal findings

This study aimed to evaluate the combined impact of MSAF and SGA on delivery outcomes. The main findings indicate higher risks for VE, VE due to NRFHR, intrapartum CD, CD due to NRFHR and the adverse neonatal outcome, in the presence of MSAF or SGA, and mostly when both risk factors were present.

Higher rates of operative deliveries, specifically due to NRFHR tracings, were observed in the presence of MSAF or SGA compared to controls. The risk was highest in the presence of the two. Our results are in line with previous studies. Addisu and Mekie [8], and Tolu et al.[22] both reported higher incidences of NRFHR patterns during delivery, as well as increased rates of VE and CD, among neonates with MSAF. Chelsea et al. [23], evaluating deliveries at term, concluded that SGA was a significant predictor of CD due to intrapartum fetal distress and of NICU admission.

It may be assumed that the mere diagnosis of a small fetus before birth predisposes clinicians to intervene more frequently. However, Sylvestre et al.[24] reported that fetuses in lower weight percentiles exhibit higher rates of CD due to fetal distress compared to fetuses at higher percentiles, even without a prior diagnosis of SGA. Hendrix et al.[25] found higher rates of placental vascular malperfusion at lower birthweight percentiles, even among infants classified as AGA. This suggests the possibility of an underlying placental pathophysiological mechanism contributing to the reduced tolerance for stress observed among small neonates. Building upon this theory, it is possible that MSAF heightens the stress-inducing environment by its vasoconstrictive effect on the umbilical vessels [26, 27], prompting smaller fetuses with limited reserves to display earlier and more pronounced signs of fetal distress; thereby, necessitating intervention.

This theory is supported by the increased rate of neonatal morbidity among SGA neonates with MSAF observed in our cohort. We found increased risk for the adverse composite neonatal outcome in neonates with MSAF or SGA birthweight, with the highest risk observed in SGA neonates with MSAF. Univariate analysis also revealed lower 5-min Apgar scores and higher rates of NICU admission in this group. The association between meconium and perinatal morbidity has been established,[1], [2], [3], [4], [5] with its effects attributed to inflammatory processes affecting fetal organs, oxidative stress, and fostering bacterial colonization within the amniotic sac [6, 7, 28], [29], [30], [31], [32] Prior research indicates that SGA fetuses, although considered at lower risk compared to those with FGR, have lower Apgar scores and higher incidences of respiratory distress, necrotizing enterocolitis, sepsis, and perinatal mortality compared to AGA fetuses [33, 34]. Additionally, some may exhibit neurodevelopmental delays later in life [35].

Steer et al. [36] conducted a case–control study evaluating the interaction of fetal heart rate abnormalities, FGR, MSAF and tachysystole, in relation to labor outcomes, and found that their integration gave the best indication of adverse outcomes during labor. They conclude that accurate risk assessment during labor should evaluate fetal heart rate abnormalities in the context of additional intrapartum risk factors. Our findings are consistent this previous report, highlighting the increased neonatal risk associated with the presence of both SGA birthweight and MSAF during delivery.

As expected, pregnancies with SGA and MSAF were characterized by higher rates of nulliparity, smoking, hypertensive disorders, and lower BMI [37], [38], [39] Notably, the higher rate of oligohydramnios observed in that group could be a marker of mild placental insufficiency, contributing to intrapartum stress. A previous study revealed that SGA with oligohydramnios significantly increases the likelihood of NICU admission, but other neonatal morbidities were not observed [40]. All the confounders mentioned above were evaluated in our logistic regression model, assuring independent associations between SGA and MSAF to the outcomes evaluated. Higher rates of labor induction also characterized the SGA MSAF group, reflecting our departmental protocol of inducing labors in pregnancies diagnosed with SGA at 38–39 weeks. Prominently, a history of CD was less common in the SGA MSAF group. These two variables were also handled in the multivariable analysis.

### Clinical Implications

The present study reveals that the combination of MSAF and SGA is a major intrapartum risk factor, increasing the rates of maternal and neonatal adverse outcomes. Clinicians should be aware of this dangerous combination during their intrapartum risk assessment and manage labor accordingly, aiming for earlier fetal extraction if there are signs of fetal distress, or if the labor does not progress appropriately. They should also be aware that SGA fetuses, even when not considered to have FGR, exhibit lower ability to cope with intrapartum stress compared to AGA fetuses, and should adjust their management strategies accordingly.

### Strengths and limitations

The main strengths of this study include the large cohort and novel evaluation, assessing a question which was not addressed previously. The detailed documentation allowed us to assemble a stratified risk profile for all study groups. Data were retrieved from a single medical center, operating under uniform medical protocols and diagnostic tools, creating a relatively homogenous cohort. The principal investigator manually reviewed the medical records of all fetuses with weights below the tenth percentile, excluding pregnancies complicated by FGR, which further contributed to the cohort's homogeneity, allowing us to evaluate the role of fetal weight in the absence of other negative prognostic factors. This study is not free of limitations, including its retrospective design and thus, its lack of some information, including data about long-term neonatal outcomes. Therefore, only short-term outcomes were analyzed. Information regarding the timing of meconium during labor was also missing and perhaps affected obstetric outcomes [41]. Decisions regarding the need for interventions for fetal extraction were influenced by the clinician's judgment, even when operating under departmental protocol. Since some of the SGA birthweights were only diagnosed postnatally, we cannot fully exclude the possibility of abnormal Doppler findings in the neonates who were diagnosed postpartum.

## Conclusions

Deliveries complicated by the co-occurrence of MSAF and SGA carry elevated obstetric risks compared to those involving either MSAF or SGA alone. These cases are associated with higher incidences of adverse neonatal outcomes, as well as increased rates of intrapartum CD and VE, particularly in response to NRFHR patterns. Clinicians should incorporate this elevated risk into their assessments and manage labor accordingly.

## Data Availability

Data will be made available from the corresponding author upon reasonable request.
